# Supplementary material for: Strengthening health service delivery and governance through institutionalizing ‘Urban Health Atlas’—A geo-referenced Information Communication and Technology tool: Lessons learned from an implementation research in three cities in Bangladesh
Source: PLoS One. 2024 Jan 25;19(1):e0266581. doi: 10.1371/journal.pone.0266581 (PMC10810507; doi:10.1371/journal.pone.0266581)
Supplement: S1 Appendix — (DOCX) [file pone.0266581.s001.docx]

**Appendix 1:** **Consolidated criteria for reporting qualitative studies (COREQ): 32-item checklist**

| **No** | **Item** | **Guide questions/description** |  |
| --- | --- | --- | --- |
| **Domain 1: Research team and reflexivity** | | | |
| Personal Characteristics | |  |  |
| 1. | Interviewer/facilitator | Which author/s conducted the interviews? | p. 12 |
| 2. | Credentials | What were the researcher's credentials? *E.g. PhD, MD* | Title p. 1, p.12 |
| 3. | Occupation | What was their occupation at the time of the study? | Title p. 1 |
| 4. | Gender | Was the researcher male or female? | SS, The PI (First Author) and AA (Senior Author) were females |
| 5. | Experience and training | What experience or training did the researcher have? | Graduate level p. 12 |
| Relationship with participants | |  |  |
| 6. | Relationship established | Was a relationship established prior to study commencement? | p. 11 |
| 7. | Participant knowledge of the interviewer | What did the participants know about the researcher? e*.g. personal goals, reasons for doing the research* | p. 11 |
| 8. | Interviewer characteristics | What characteristics were reported about the interviewer/facilitator? e.g. *Bias, assumptions, reasons and interests in the research topic* | Inferred on p. 10-11 |
| **Domain 2: study design** | | | |
| Theoretical framework | |  |  |
| 9. | Methodological orientation and Theory | What methodological orientation was stated to underpin the study? *e.g. grounded theory, discourse analysis, ethnography, phenomenology, content analysis* | Framework method – form of thematic analysis p. 13 |
| Participant selection | |  |  |
| 10. | Sampling | How were participants selected? *e.g. purposive, convenience, consecutive, snowball* | Opportunistic & snowball Purposive sampling; p. 8 |
| 11. | Method of approach | How were participants approached? e*.g. face-to-face, telephone, mail, email* | Face to face, telephone & email |
| 12. | Sample size | How many participants were in the study? | 30 |
| 13. | Non-participation | How many people refused to participate or dropped out? Reasons? | None |
| Setting | |  |  |
| 14. | Setting of data collection | Where was the data collected? e*.g. home, clinic, workplace* | Workplace |
| 15. | Presence of non-participants | Was anyone else present besides the participants and researchers? | No |
| 16. | Description of sample | What are the important characteristics of the sample? *e.g. demographic data, date* | p. 10-11 |
| Data collection | |  |  |
| 17. | Interview guide | Were questions, prompts, guides provided by the authors? Was it pilot tested? | p. 12 (Appendix 2) |
| 18. | Repeat interviews | Were repeat interviews carried out? If yes, how many? | No |
| 19. | Audio/visual recording | Did the research use audio or visual recording to collect the data? | Both |
| 20. | Field notes | Were field notes made during and/or after the interview or focus group? | Yes; p. 12 |
| 21. | Duration | What was the duration of the interviews or focus group? | 30-45 minutes  p. 12 |
| 22. | Data saturation | Was data saturation discussed? | Yes; p. 8 |
| 23. | Transcripts returned | Were transcripts returned to participants for comment and/or correction? | No |
| **Domain 3: analysis and finding** | | | |
| Data analysis | |  |  |
| 24. | Number of data coders | How many data coders coded the data? | Three; p. 13 |
| 25. | Description of the coding tree | Did authors provide a description of the coding tree? | Codes emanating from the framework in Figure 1 |
| 26. | Derivation of themes | Were themes identified in advance or derived from the data? | Both; p. 14 |
| 27. | Software | What software, if applicable, was used to manage the data? | ATLAS-ti (version-7.5.7); p. 13 |
| 28. | Participant checking | Did participants provide feedback on the findings? | Yes, through the Technical Advisory Group (TAG); p. 16 |
| Reporting | |  |  |
| 29. | Quotations presented | Were participant quotations presented to illustrate the themes / findings? Was each quotation identified? e*.g. participant number* | Yes, in paper. |
| 30. | Data and findings consistent | Was there consistency between the data presented and the findings? | Yes |
| 31. | Clarity of major themes | Were major themes clearly presented in the findings? | Yes |
| 32. | Clarity of minor themes | Is there a description of diverse cases or discussion of minor themes? | Yes |
