# Supplementary material for: Strengthening health service delivery and governance through institutionalizing ‘Urban Health Atlas’—A geo-referenced Information Communication and Technology tool: Lessons learned from an implementation research in three cities in Bangladesh
Source: PLoS One. 2024 Jan 25;19(1):e0266581. doi: 10.1371/journal.pone.0266581 (PMC10810507; doi:10.1371/journal.pone.0266581)
Supplement: S2 Appendix — (DOCX) [file pone.0266581.s002.docx]

# Annex 2: Guideline for Key Informant Interview (KII) on Task Review

*[General instructions: During interview, interviewer should start by introducing self, the purpose and the expected time (1 hour). If agreeable to interview, continue with the consent form and get that signed. Request to record interview and make sure the tape recorder is switched on to the start of the interview. If denied, take notes.]*

**Background information** *[Note: to be filled in by note taker]*

- Identification no:
- Gender:
- Level of education:
- Type of the respondent (Government official/NGO programme manager/ MNCH service provider/ private sector/recipient/ others)
- Organization:
- Current position:

**Review of general tasks (job description)**

1. Let us brainstorm and list freely all tasks required to perform your job successfully.
   - What are your major tasks?
   - What are the competencies you need to perform these tasks?
   - Who else assists you in these tasks?
   - Please explain what working conditions are needed to successfully perform your job?

**Identifying specific tasks using Information Communication and Technology (ICT)**

*[Note: Particular individuals and their tasks needs be considered while asking questions.]*

1. What type of tasks do you perform that are related to ICT?
   - How does ICT help you to perform these tasks?
     - What else is involved in this task?
     - What information do you need to perform the job task efficiently?
     - What competencies are needed to perform ICT assisted tasks?

3. What types of tasks do you do that need information related to health facilities (their location, services provider, procurement information, referral chains, human resources etc.) etc.?

- How can UHA help you to perform your job more efficiently:
  - - Informing any committee on regular basis
    - Licensing
    - Allocation of HR
    - Medicine distribution etc.

**Annex 3: Guideline for Key Informant Interview (KII) on ICT contribution in policy making**

*[General instructions: During interview, interviewer should start by introducing self, the purpose and the expected time (1 hour). If agreeable to interview, continue with the consent form and get that signed. Request to record interview and make sure the tape recorder is switched on to the start of the interview. If denied, take notes.]*

**Background information** *[Note: to be filled in by note taker]*

- Identification no:
- Gender:
- Level of education:
- Type of the respondent (Government official/NGO programme manager/ MNCH service provider/ private sector/recipient/ others)
- Organization:
- Current position:

**Policy position**

1. How long have you been working in your current position?

- What is your background and current role in policy/program process?

**Policy making process related to ICT**

1. Please explain the process/mechanism of health policy making within the systems.

- Who makes the decisions in policy/program? (probe about actors, factors, and other influences like political, financial resources, incentives)
- What is your opinion regarding the integration of Information Communication Technology (ICT) in health policy?
- What other policies effect the integration of ICT in health?
- What are your views on importance of ICTs in planning and decision making processes?

**Challenges in integrating ICT**

1. According to your opinion, what are the barriers and challenges in incorporation of ICT in health policy making in Bangladesh?

- How to overcome these challenges?
- How can the incorporation of ICT in health systems become sustainable?

**Institutionalization of geo-referenced health facility information**

1. How much geo-referenced health facility information is being used for policy making?
2. What is your opinion regarding incorporation of geo-referenced health facility information in health policy?

- To what extent you think it will be helpful in planning and decision making?
- What are your suggestions/recommendations?

**Annex 3:** In-depth Interview (IDI) on user needs and reflections

*[General instructions: During interview, interviewer should start by introducing self, the purpose and the expected time (1 hour). If agreeable to interview, continue with the consent form and get that signed. Request to record interview and make sure the tape recorder is switched on to the start of the interview. If denied, take notes.]*

**Background information** *[Note: to be filled in by note taker]*

- Identification no:
- Gender:
- Level of education:
- Type of the respondent (Government official/NGO programme manager/ MNCH service provider/ private sector/recipient/ others)
- Organization:
- Current position:

**Experience of using ICT tools**

1. How frequently do you use ICT tools (e.g. computer/tab/smart phone)?

- Where would you put yourself in terms of using these tools?

Please rank on a 10 point scale: 0 – 1 – 2 – 3 – 4 – 5 – 6 – 7 – 8 – 9 – 10.

- Why are you on that position?

**Experience of using ICT tools for health**

1. Have you ever used any such tool in your work? Can you please tell us about that/those with some examples? [e.g. punch ID while arriving at work, use tabs for surveys, fill in various online reports, log into different website]
2. Have you used any other types of tools? Please explain in details. (e.g. monthly monitoring tool, procure equipment, HMIS)

- Any challenges faced while using? (e.g. difficulty in monitoring, system down)
- How did you overcome those challenges?]

1. Have you ever used DHIS-2 for your work purpose?

- Where would you put yourself in terms of using DHIS-2?

Please rank on a 10 point scale: 0 – 1 – 2 – 3 – 4 – 5 – 6 – 7 – 8 – 9 – 10.

- How has this tool changed the way you work? (e.g. benefitted your work, positively impacted)
- Have you ever used any other ICT tools for your work besides DHIS2 (e.g. Open MRS etc)?

**Experience of using online maps**

1. Have you ever used any online map in any of these gadgets?

- How did you use online maps?
- Where would you put yourself in terms of using online maps?

Please rank on a 10 point scale: 0 – 1 – 2 – 3 – 4 – 5 – 6 – 7 – 8 – 9 – 10.

- Why are you on that position in terms of using online maps?

**Experience of using Urban Health Atlas (UHA)**

***Personal use***

*[Note: Using health facility data from 7 city corporations in Bangladesh, we developed a web-based interactive data visualization tool with GIS functionality named the Urban Health Atlas (UHA). We can show the tool if possible.]*

1. Do you know about the Urban Health Atlas (UHA)? If yes, have you ever used the UHA?

- Why did you use it? How did you use it?
- How far do you think Urban Health Atlas (UHA) tool will be useful for your work?
  - Please rank on a 10 point scale: 0 – 1 – 2 – 3 – 4 – 5 – 6 – 7 – 8 – 9 – 10. *[Usability]*
  - Why did you rank yourself at that position?
- Do you find this tool easily understandable for your own work purposes?
  - Please rank on a 10 point scale: 0 – 1 – 2 – 3 – 4 – 5 – 6 – 7 – 8 – 9 – 10. *[Understandability]*
  - Why did you rank yourself at that position?
- How frequently do you use the tool for your work?
  - Please rank on a 10 point scale: 0 – 1 – 2 – 3 – 4 – 5 – 6 – 7 – 8 – 9 – 10. *[Utility]*
  - Why did you rank yourself at that position?
- How confident do you feel in using the tool?
  - Please rank on a 10 point scale: 0 – 1 – 2 – 3 – 4 – 5 – 6 – 7 – 8 – 9 – 10.
  - Why did you rank yourself at that position?

***Use of UHA for MNCH decision making***

1. To what extent would UHA be helpful in MNCH decision making?

- What is your opinion regarding application of UHA to aid in urban MNCH planning? (e.g. accountability)
- What do you think about the feasibility of this tool for strengthening MNCH services in the country?

***Suggestions on institutionalizing UHA***

1. What could be added or done to the UHA for better use in your work? How could use of the tool be encouraged?

- What problems/hurdles you think you might face while using the UHA?
- What kinds of training would be helpful in your opinion for better usability of UHA?
- How do you want the training to be conducted?

*P****otential Users of UHA***

1. Who do you think will be the frequent users of this tool?

- How do you think they could use it?

*[Finish the interview by thanking the participant.]*

# Annex 4: In-depth Interview (IDI) on user experiences

*[General instructions: During interview, interviewer should start by introducing self, the purpose and the expected time (1 hour). If agreeable to interview, continue with the consent form and get that signed. Request to record interview and make sure the tape recorder is switched on to the start of the interview. If denied, take notes.]*

**Background information** *[Note: to be filled in by note taker]*

- Identification no:
- Gender:
- Level of education:
- Type of the respondent (Government official/NGO programme manager/ MNCH service provider/ private sector/recipient/ others)
- Organization:
- Current position:

**Experience on using Urban Health Atlas**

1. Let us discuss your experience of using UHA tool during/after the training.

- How frequently do you use the UHA tool in you work? For what purposes?
- How far do you think Urban Health Atlas (UHA) tool will be useful for your work?
- Please rank on a 10 point scale: 0 – 1 – 2 – 3 – 4 – 5 – 6 – 7 – 8 – 9 – 10. *[Usability]*
- Why did you rank yourself at that position?
- Do you find this tool easily understandable for your own work purpose?
  - Please rank on a 10 point scale: 0 – 1 – 2 – 3 – 4 – 5 – 6 – 7 – 8 – 9 – 10. *[Understandability]*
  - Why did you rank yourself at that position?
- How frequently do you use the tool for your work?
- Please rank on a 10 point scale: 0 – 1 – 2 – 3 – 4 – 5 – 6 – 7 – 8 – 9 – 10. *[Utility]*
- Why did you rank yourself at that position?
- How confident do you feel in using the tool?
- Please rank on a 10 point scale: 0 – 1 – 2 – 3 – 4 – 5 – 6 – 7 – 8 – 9 – 10.
- Why did you rank yourself at that position?

**Challenges of using UHA**

1. Now let us talk about the challenges you encountered while using the UHA.

- What problems/hurdles did you face while using the UHA?
- How did you overcome these?
- Was there any issue that you could not resolve? What was that?
- What are your suggestions for improving the tool to solve such issues?

**Successes of using UHA**

1. What is your opinion regarding the UHA as a means of producing credible data for strategic planning in MNCH services? [Better indicators for strategic planning]
   - What are your thoughts about managers being able to act on this information?
   - How did it change the way people work?
2. Did information generated by UHA change decisions regarding MNCH service planning and referral? [Better indicators for strategic planning]

- If yes, how beneficial was the change?

1. What is your opinion regarding the UHA leading to more efficient operations of the MNCH services? [Better day-to-day decision-making]

- Was there any measurable impact on outcome indicators?

1. Do health facility data furnished by UHA accurately reflect reality? [Better control and oversight]

- How has this data been useful in health systems planning and oversight/management?

1. Comparing how people spent their time before using UHA and how they spend it since use of UHA, what are the differences? [Reduced administrative burden]

- Is there any reduced administrative burden associated with implementation of UHA?

*Probe: monthly monitoring, monthly reporting, equipment procurement.*

**Suggestions**

1. How do you think the tool can be improved for practical use?

- What changes you recommend on this tool to make it handier?
- What other kinds of training should be helpful in your opinion for better usability of UHA?
- What will be your recommendations to other people regarding use of this UHA for MNCH service decision making?
